# GABALAGEN Alleviates Stress-Induced Sleep Disorders in Rats

**DOI:** 10.3390/biomedicines12122905

**Published:** 2024-12-20

**Authors:** Hyun-Jung Park, Sung Ja Rhie, Woojin Jeong, Kyu-Ri Kim, Kyoung-Min Rheu, Bae-Jin Lee, Insop Shim

**Affiliations:** 1 Department of Food Science and Biotechnology, Kyonggi University, Suwon 16227, Republic of Korea; phj1116@gmail.com; 2 Department of Beauty Design, Halla University, Wonju 26404, Republic of Korea; sjlee@halla.ac.kr; 3 Department of Physiology, College of Medicine, Kyung Hee University, Seoul 02447, Republic of Korea; as84315077@gmail.com (W.J.); kyuri_kim@khu.ac.kr (K.-R.K.); 4 Marine Bioprocess Co., Ltd., Busan 47281, Republic of Korea; kim.rheu@gmail.com (K.-M.R.); hansila82@hanmail.net (B.-J.L.)

**Keywords:** GABA_A_ receptor, electroencephalography (EEG), electro foot shock (EFS), sleep, orexin

## Abstract

(1) Background: Gamma-aminobutyric acid (GABA) is an amino acid and the primary inhibitory neurotransmitter in the brain. GABA has been shown to reduce stress and promote sleep. GABALAGEN (GBL) is the product of fermented fish collagen by Lactobacillus brevis BJ20 and Lactobacillus plantarum BJ21, naturally enriched with GABA through the fermentation process and characterized by low molecular weight. (2) Methods: The present study evaluated the GABA_A_ affinity of GBL through receptor binding assay. The sedative effects of GBL were investigated through electroencephalography (EEG) analysis in an animal model of electro foot shock (EFS) stress-induced sleep disorder, and then we examined the expression of orexin and the GABA_A_ receptor in the brain region using immunohistochemistry and an enzyme-linked immunosorbent assay (ELISA). (3) Results: We found that on the binding assay, GBL displayed high affinity to the GABA_A_ receptor. Also, after treatment with GBL, the percentage of the total time in rapid eye movement (REM) and non-rapid eye movement (NREM) sleep was significantly and dose-dependently increased in EFS-induced rats. Consistent with behavioral results, the GBL-treated groups showed that the expression of GABA_A_ receptor immune-positive cells in the VLPO was markedly and dose-dependently increased. Also, the GBL-treated groups showed that the expression of the orexin-A level in LH was significantly decreased. (4) Conclusions: GBL showed efficacy and potential to be used as an anti-stress therapy to treat sleep deprivation through the stimulation of GABA_A_ receptors and the consequent inhibition of orexin activity.

## 1. Introduction

Post-traumatic stress disorder (PTSD) is a chronic mental disorder caused by uncontrollable exposure to real or threatening death or serious injury, characterized by prominent sleep disorders including recurrent and stereotypical nightmares and insomnia [1,2,3]. Sleep disorder is characterized by difficulties in initiating or maintaining sleep, non-restorative sleep, or poor-quality sleep [4,5]. There has been much research into the effects of stress on sleep in rodents [6]. The primary stressors used in previous studies were restraint stress and mild electric shock, with few reports using other methods [7,8,9]. These sleep disturbances are a prominent feature, and potentially even a hallmark of PTSD [6,10].

Orexin is a neuropeptide whose main function is the regulation of the sleep–wake cycle. Orexin regulates a wide variety of important bodily functions, such as eating behavior, stress response, reward processing, mood, and cognition [11,12,13,14,15]. The activity of orexin is regulated by changes in receptors of the inhibitory neurotransmitter, gamma-aminobutyric acid (GABA). Previous studies have shown that orexin neurons are regulated by GABA receptor agonists (e.g., baclofen) and antagonists (e.g., bicuculline) [16,17,18,19,20,21]. Also, previous studies reported daytime sleepiness, narcolepsy, rapid eye movement (REM) sleep abnormalities, and, in some cases, an association between orexin deficiency narcolepsy [11,12,13,14]. The types of drugs commonly used for the treatment of sleep disorders include GABA type A (GABA_A_)-benzodiazepine (BZD) receptor agonists, selective serotonin reuptake inhibitors, melatonin receptor agonists, antidepressants, and antihistamines [22,23,24]. The mechanism by which benzodiazepines such as diazepam (DZP) enhance the GABA receptor function is called allosteric regulation. In this sense, allosteric means that DZP binds at a site distinct from GABA_A_ agonist binding site. Structure–function studies have indeed demonstrated that the DZP binding site is distinct from the GABA binding site [25]. GABA is an amino acid that occurs naturally in the human body, and it plays a crucial role in maintaining mental health. It is involved in various physiological processes, such as increasing brain protein synthesis, elevating growth hormone concentration, lowering high blood pressure, regulating diabetes, and facilitating diuresis. GABA has a calming effect and is potentially useful in treating insomnia and depression [23,24,26]. 

GABALAGEN (GBL) is the product of fermented fish collagen by Lactobacillus brevis BJ20 (L. brevis BJ20) and Lactobacillus plantarum BJ21 (L. plantarum BJ21), naturally enriched with GABA through the fermentation process and characterized by low molecular weight, that is easily absorbed by the body. Various studies have shown that some strains of L. brevis have the ability to produce GABA from nitrogen and carbon sources [27,28,29,30]. In a previous study, it was reported that fermented seaweed, isolated from kimchi using *Lactobacillus brevis*, had a concentrated GABA content (5.56% dry weight) and antioxidant activity [31]. Furthermore, lactobacillus brevis-fermented GABA ameliorates depression-like behaviors and increases slow wave sleep [32,33]. However, research on the impact of GBL on sleep is limited despite various studies being conducted, and there is currently no conclusive understanding of the mechanisms for treating or improving sleep and related disorders.

This study evaluated the GABA_A_ affinity of GBL using a receptor binding analysis. The sedative effects of GBL were investigated using an electroencephalography (EEG) analysis in a foot shock stress (EFS) animal model, and then the expression of orexin and GABA_A_ receptors in brain regions was investigated using immunohistochemistry (IHC) and an enzyme-linked immunosorbent assay (ELISA).

## 2. Materials and Methods

### 2.1. Preparation of GBL 

GBL was obtained from Marine Bioprocess Co., Ltd. (Busan, Republic of Korea). The production process began with hydrolyzing fish collagen sourced from Geltech Co., Ltd. (Busan, Republic of Korea) at 55 ± 2 °C for 12 h using prozyme (Bisionbiochem Co., Ltd, Seoul, Republic of Korea). The manufacturing involved two successive fermentation steps using Lactobacillus brevis BJ20 (accession No. KCTC 11377BP) and Lactobacillus plantarum BJ21 (accession No. KCTC 18911P). To prepare the seed medium, 3% yeast extract (Choheung, Ansan, Republic of Korea), 1% glucose (Choheung, Ansan, Republic of Korea), 1% L-glutamic acid (Samin Chemical, Siheung, Republic of Korea), and 95% water were mixed and sterilized at 121 °C for 15 min. The sterilized medium was then inoculated with 0.002% L. brevis BJ20 and 0.002% L. plantarum BJ21, and the cultures were grown separately at 37 °C for 24 h. In the first fermentation stage, 10% (*v*/*v*) of the L. brevisBJ20 seed culture was added to the fermentation medium, which consisted of 2% yeast extract, 0.28% glucose, 29% hydrolyzed fish collagen (Geltech Co., Ltd., Busan, Korea), 5.5% L-glutamic acid (Samin Chemical, Siheung, Republic of Korea), and 63.22% water. The fermentation was carried out at 37 °C for 24 h. Following this, 10% (*v*/*v*) of the L. plantarum BJ21 seed culture was introduced, and the mixture was fermented for an additional 24 h at the same temperature. The final fermentation medium was sterilized and spray-dried to produce the GBL powder.

### 2.2. GABA_A_ Receptor Binding Assay

GABA receptors were obtained from rat brain tissue. Protein chips were obtained from proteogen, and Cy5-labeled muscimol was obtained from peptron. Muscimol is a GABA_A_ agonist that binds at the interface between the α1 and β2 subunits. The stock buffer of GABA receptors consisted of 50 mM Tris-HCl (pH 7.4), 0.5 mM EDTA, 10 mM MgCl2, and 10% sucrose. The GABA receptor binding assay buffer, which served as a substrate to capture proteins at pH 7.4, consisted of 50 mM Tris-HCl, 10 mM MgCl, 1 mM EDTA, and 0.1% bovine serum albumin (BSA), and the receptors (50 µg/mL) were immobilized on the protein chips for 16 h at 4 °C. After double washing with 0.05% phosphate-buffered saline containing 0.2% Triton X-100 (PBST) for 10 min and drying with nitrogen gas (N2), the protein chip underwent a blocking step with 3% BSA for 1 h at room temperature. After washing three times with PBST and drying, Cy5-labeled muscimol (500 µM, containing 30% glycerol in PBS) and GBL (using 30% glycerol buffer in PBS) were applied to the protein chip and incubated at 37 °C for 1 h. The protein chip was then rinsed with PBST and DW and dried under N2 gas flow. GBL, dissolved in ethanol and diluted to the desired concentrations using PBS, covered the concentration range from 1000 µM to 15.625 µg/mL. Muscimol was used as a negative control. GBL was used to evaluate the sedative effect.

### 2.3. Animals

**Figure 1:**
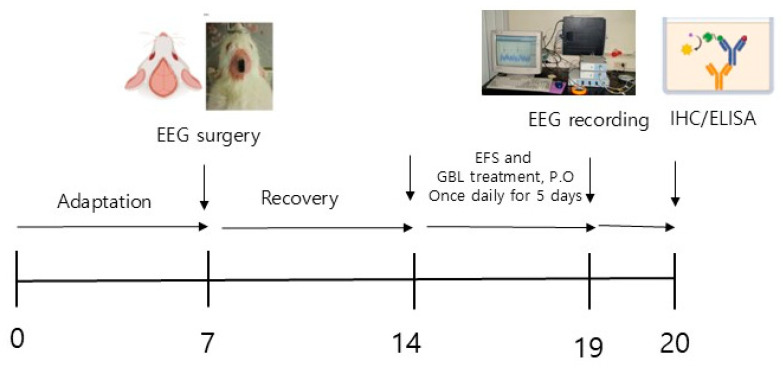
Experimental setup.

Adult male Sprague Dawley rats were obtained from Samtaco (Osan-si, Gyeonggi-do, Republic of Korea). The animals were housed in a climate-controlled environment with a temperature in the range of 20–25 °C and humidity in the range of 45–65% after a 12 h light/12 h dark cycle (lights on at 8:00 AM). Food and water were provided ad libitum during the study. The animals were handled and treated according to the guidelines outlined by the National Institute of Toxicology and the U.S. Food and Drug Administration (FDS) in accordance with the Standard for the Care and Use of Laboratory Animals (Approval Number: KHUASP[SE]-14-051). The experimental setup is shown in Figure 1.

### 2.4. Surgery

The subjects were divided into five groups: untreated, naïve normal (Nor; *n* = 7), vehicle treated control (Con; *n* = 8), the low-dose group treated with 100 mg/kg of GBL (GBL_L; *n* = 8), the high-dose group treated with 250 mg/kg of GBL (GBL_H; *n* = 7), and the group treated with 10 mg/kg of diazepam as a positive control (PC; *n* = 7). Electroencephalography (EEG) electrodes were surgically implanted to facilitate polygraphic recordings according to the guidelines outlined in the Paxinos and Watson stereotaxic atlas [34]. Surgical anesthesia was intraperitoneally induced with pentobarbital (40 mg/kg). The rats were then chronically implanted with head mount. The transmitter body was placed subcutaneously behind the scapula, off the midline, and secured to the skin with three sutures. The electrodes mounted on the skull were fixed with screws and dental cement. All surgical interventions were performed using stereotaxic methodology in an aseptic environment. After surgery, each rat was allowed a 7-day recovery period in a separate transparent enclosure. After electroencephalography (EEG) surgery, animals received electric foot shocks (EFSs) once daily for 5 days. EFSs were administered randomly according to the following conditions: frequency = 10 times for 3 s; intensity = 3 mA; duration = 5 min.

### 2.5. EEG Recording

After the recovery phase, rats were acclimated to the recording setup prior to testing. GBL_L, GBL_H, and PC were prepared in 0.9% saline (GBL_L concentration = 100 mg/kg; GBL_H concentration = 250 mg/kg; as a PC, DZP concentration = 10 mg/kg) and administered orally for 5 consecutive days before the start of EEG recording. Oral administration of saline, GBL_L, GBL_H, and PC were performed 10 min before the EEG recording session. Recordings started at 8:00 PM, capturing 12 h of EEG and activity in all subjects. Cortical EEG signals were amplified (×100), filtered through a low-pass filter at a rate of 100 Hz, digitized at a sampling rate of 200 Hz, and recorded using a PAL-8200 data acquisition system from Pinnacle Technologies. Recordings were performed at a chart speed of 25 mm/s. SleepSign Ver. 3 Software (Kisei Comtec, Nagano, Japan) automatically classifies sleep–wake states into three categories: wake, rapid eye movement (REM) sleep, and nonrapid eye movement (NREM) sleep. Epoch length can be selected among the following periods: 4, 5, 8, 10, 20, 30, and 60 s. SleepSign calculates average FFT spectrums of each sleep/wake stage by each time bin and time range. Sleep latency was defined as the time from sample administration to the onset of the initial uninterrupted NREM sleep episode lasting at least 2 min and lasting 4 s or more that was not recorded as NREM sleep.

### 2.6. Immunohistochemistry

After transcranial perfusion, rat brains were cleared with 4% formaldehyde solution (Sigma-Aldrich Co., St. Louis, MO, USA), postfixed in the same fixative for 24 h, and then placed in phosphate-buffered saline containing 20% sucrose for 72 h. Coronal sections 30 µm in thickness were cut using a cryostat microtome (CM1850UV; Leica Microsystems, Wetzlar, Germany), stored at −20 °C, and then processed immunohistochemically as free-floating sections. Sections were washed three times with PBST. Antibodies targeting GABA_A_ receptors (Abcam, Cambridge, MA, USA) were diluted in a ratio of 1:800. Sections were incubated at 4 °C for 12 h with continuous shaking. After rinsing with PBST, sections were incubated with biotinylated goat anti-rabbit antibody (Vector Laboratories, Inc., Burlingame, CA, USA) at a 1:200 dilution in PBST with 2% *v*/*v* normal goat serum for 2 h at room temperature. Sections were then exposed to avidin–biotin–peroxidase complex reagent (Vector Laboratories) for 2 h at room temperature. After additional rinsing with PBST, tissues were developed using a DAB substrate kit (Vector Laboratories). Final steps included mounting sections on slides, air-drying, and coverslipping for microscopic observation.

### 2.7. ELISA (Enzyme-Linked Immunosorbent Assay)

ELISA was used to assess the expression level of orexin-A in LH using an ELISA kit (Antibody-Online Inc. Pottstown, PA, USA) according to the manufacturer’s instructions. Briefly, all samples were tested in duplicate per mouse. Prepared tissue samples were diluted using 96-well strip plates, standards (liquid; ×2), detection reagent A (120 µL), detection reagent B (120 µL), TMB substrate (9 mL), wash buffer (20 mL), 96-well plate sealer (x4), standard diluent (20 mL), assay diluent A (6 mL), diluent B (6 mL), and stop solution (6 mL). After preparation of all reagents and setting up of dilution samples, standards and samples were diluted, respectively. After the reaction was completed, samples were analyzed using a microplate reader (Model 680, Bio-Rad Laboratories, Inc. Hercules, CA, USA) and measured immediately at 450 nm. The optical density values of the standard (*X*-axis) were plotted against the log of the concentration of the standard (*Y*-axis), and the concentration of orexin-A was calculated.

### 2.8. Statistical Analysis

All statistical analyses were performed using SMS (IBM^®^ SPSS^®^ Statistics Ver. 23, Chicago, IL, USA). Behavioral data were analyzed using a one-way analysis of variance (ANOVA) for multiple comparisons. Tukey’s post hoc test was used to identify significant differences between groups. The significance level was set at *p* < 0.05.

## 3. Results

### 3.1. GABA_A_ and 5-HT2c Receptor Binding Assay

**Figure 2:**
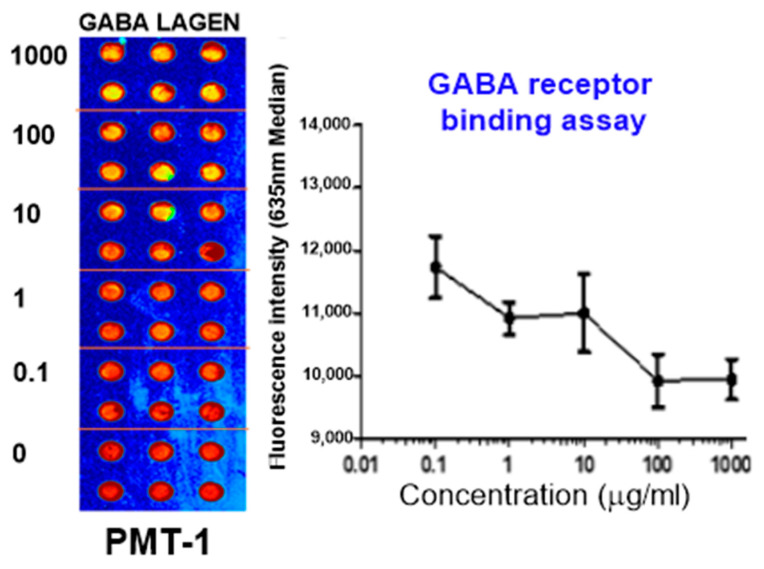
The binding activities of GBL to the GABA_A_ receptor.

As shown in Figure 2, the binding activities of GBL to the GABA_A_ receptor, indicative of its role as a GABA_A_ receptor agonist, were substantiated. The IC50 value for GBL in this context was measured to be 31.5 µg/mL (Figure 2).

### 3.2. Effect of GBL on EEG Sleep Architecture

**Figure 3:**
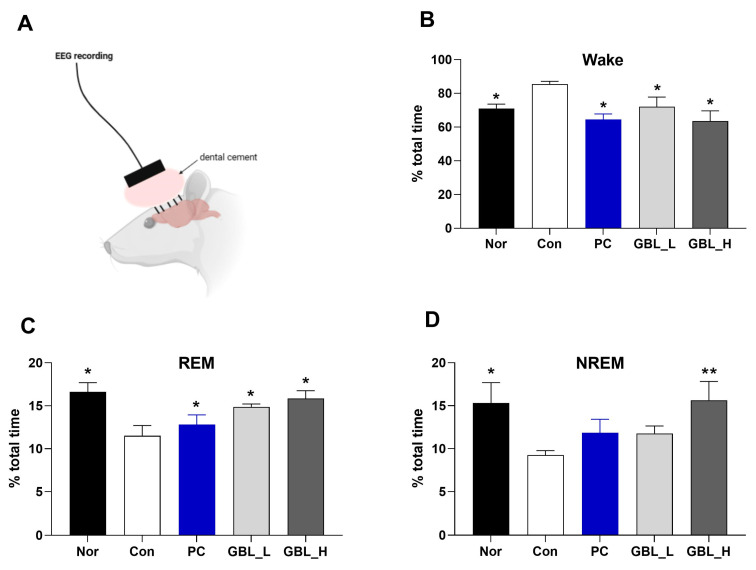
The effect of GBL on the EEG sleep architecture and profile. EEG recording apparatus (**A**) and changes in the percentages of time spent awake (**B**), in REM (**C**), and in NREM (**D**) are shown. The values are presented as means ± S.E.M. * *p* < 0.05 and ** *p* < 0.01 vs. Con group. The subjects were divided into five groups: normal (Nor; *n* = 7), control (Con; *n* = 8), the low-dose group treated with 100 mg/kg of GBL (GBL_L; *n* = 8), the high-dose group treated with 250 mg/kg of GBL (GBL_H; *n* = 7), and the group treated with 10 mg/kg of diazepam as a positive control (PC; *n* = 7).

The effect of GBL on EEG sleep architecture was investigated. There are significant differences among groups (F_4,32_ = 7.2; *p* < 0.01; Figure 3B). The percentages of the time spent awake in the Con group was markedly increased compared to the Nor group (*p* < 0.05). However, the GBL and PC-treated groups showed markedly decreased percentages of time spent awake compared to the Con group (*p* < 0.05). Also, the Con group had a markedly decreased REM sleep percentage (F_4,32_ = 6.4; *p* < 0.01; Figure 3C) and NREM sleep percentage (F_4,32_ = 4.0; *p* < 0.05; Figure 3D) compared to the Nor group. However, after the treatment with GBL, the percentage of the total time of REM and NREM significantly and dose-dependently increased compared to the Con group (REM: *p* < 0.05, Figure 3C; NREM: *p* < 0.01, Figure 3D).

### 3.3. The Effect of GBL on the Number of GABA_A_ Receptor Positive Cells in the Ventrolateral Preoptic Nucleus (VLPO)

**Figure 4:**
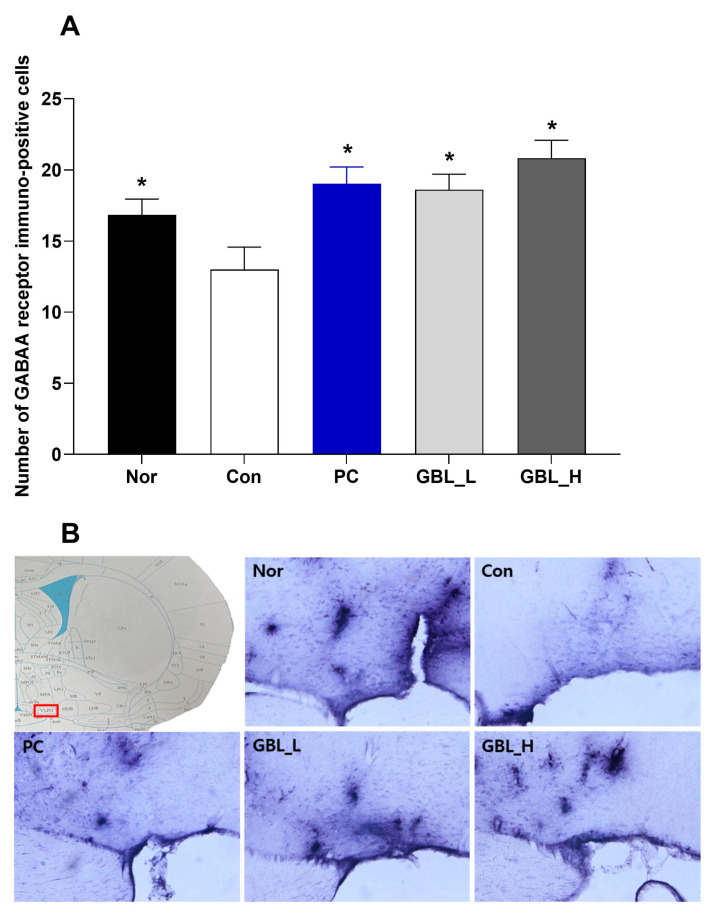
The effect of GBL on the number of GABA_A_ receptor positive cells in the ventrolateral preoptic nucleus (VLPO). (**A**) Quantification of GABA_A_ receptor positive cells in the VLPO. (**B**) Photomicrographs illustrating GABA_A_ receptor positive cells in the VLPO. The values are presented as the means ± S.E.M. * *p* < 0.05 vs. the Con group. The subjects were divided into five groups: normal (Nor; *n* = 7), control (Con; *n* = 8), the low-dose group treated with 100 mg/kg of GBL (GBL_L; *n* = 8), the high-dose group treated with 250 mg/kg of GBL (GBL_H; *n* = 7), and the group treated with 10 mg/kg of diazepam as a positive control (PC; *n* = 7).

We measured the GABA_A_ receptors’ immunoreactivity in the VLPO (F_4,32_ = 6.8, *p* < 0.05, Figure 4A,B). The numbers of GABA_A_ receptor immuno-positive cells were significantly decreased in the Con group (*p* < 0.05). However, the GBL and PC-treated groups showed that the expression of GABA_A_ receptor immune-positive cells was markedly increased compared to the Con group (*p* < 0.05).

### 3.4. The Effect of GBL on the Orexin Level in the Lateral Hypothalamus (LH)

**Figure 5:**
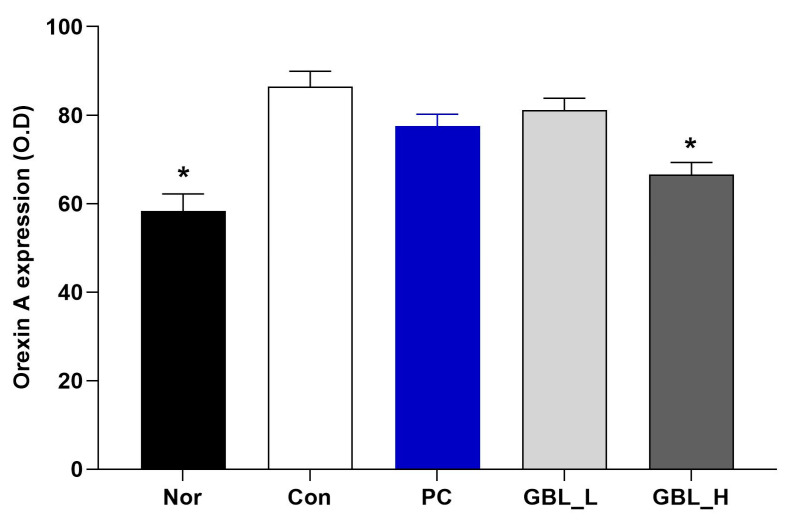
The effect of GBL on orexin A expression in the lateral preoptic nucleus (LH). The values are presented as the means ± S.E.M. * *p* < 0.05 vs. Con group. The subjects were divided into five groups: normal (Nor; *n* = 7), control (Con; *n* = 8), the low-dose group treated with 100 mg/kg of GBL (GBL_L; *n* = 8), the high-dose group treated with 250 mg/kg of GBL (GBL_H; *n* = 7), and the group treated with 10 mg/kg of diazepam as a positive control (PC; *n* = 7).

We measured the orexin-A level in the LH (F_4,32_ = 12.9; *p* < 0.001; Figure 5). The level of orexin-A was significantly increased in the Con group (*p* < 0.05). However, the GBL-treated groups showed that the expression of the orexin-A level in the LH was significantly decreased compared to the Con group (*p* < 0.05).

## 4. Discussion

The present study showed that in binding assays, GBL showed binding affinity for GABA_A_ receptors (IC50 value, 31.5 µg/mL). Additionally, after treatment with GBL, the fraction of the total time in REM and NREM significantly increased in a dose-dependent manner compared to the Con group. Consistent with the behavioral results, the GBL-treated group showed a significant dose-dependent increase in the expression of GABA_A_ receptor immunopositive cells in the VLPO compared to the Con group. Additionally, the GBL-treated group showed a significant decrease in orexin levels in the LH compared to the Con group. GBL has shown efficacy and the potential to be used as an anti-stress therapy to treat sleep deprivation through the antagonism of GABA receptor inhibitors and the subsequent inhibition of orexin activity.

Stress can have lasting negative effects on one’s health, including changes in behavior and sleep [7,8,9,35,36]. Sleep reactivity tends to cause sleep disturbances during environmental disturbances, pharmacological issues, or stressful life events [37]. As a result, individuals with highly responsive sleep systems are prone to insomnia disorders after stressors, which pose a risk of psychopathology and potentially hinder their recovery from traumatic stress [38,39,40,41,42,43,44]. Therefore, there is tremendous value in improving sleep responsiveness, creating a robust sleep system for stress exposure, and ultimately preventing insomnia and subsequent consequences. 

GBL is a type of collagen with a low molecular weight that contains high concentrations of GABA. It is ingredient which is low molecular weight fish collagen that we use to ferment that converts into even lower molecular weight collagen peptide that is easily absorbed by the body. The VLPO is composed of a diverse population of neurons and is regulated by afferent inputs from many sources of different neurotransmitters [45,46,47]. During sleep, GABAergic neurons activated in the VLPO promote sleep by inhibiting components of the ascending arousal pathway [48]. Some of these inputs have been shown to respond differently to neurotransmitters such as noradrenaline (NA) and 5-HT [1,49,50]. In fact, sleep-promoting neurons within the VLPO are generally seen to inhibit NA. In addition, previous studies have reported that the microinjection of 5-HT precursors into the hypothalamic region including the VLPO can restore prolonged sleep [51,52,53]. Previous studies have suggested that many herbal medicines enhance GABAergic signaling through interaction with GABA receptors [54,55,56,57]. Another study proved that GABA from fermented rice germ treatment reduced caffeine-induced sleep disturbance [58]. A clinical study also reported that the treatment of rice germ-derived GABA improved sleep efficacy [59]. We also found that GBL markedly increased both NREM and REM during sleep, and the GBL treatment group showed a concentration-dependent increase in the expression of GABA_A_ receptor immune-positive cells in the VLPO compared to the Con group. It is speculated that GBL treatment may modulate the NA-5-HT sleep pathway via GABA receptor activation.

Orexin neurons are characterized by wakefulness-promoting activity, reaching peak firing rates during wakefulness [60,61,62] and exhibiting the highest extracellular levels of orexin during wake [63]. The LH coordinates sleep-promoting activities, and disruptions in orexin function may upset the delicate balance between sleep and wakefulness, thereby contributing to a spectrum of sleep-related problems [64,65,66]. This emphasizes the importance of investigating the role of the LH and orexin in comprehending and addressing sleep-related disturbances [61,66]. We found that GBL decreased the orexin level in the LH region. GBL may decrease the orexin level in LH, leading to a reduction in arousal and potentially enhancing the quality of sleep.

Taken together, the current study suggests that GBL may help manage stress through the regulation of GABAergic mechanisms and orexin.

## 5. Conclusions

In summary, the present study showed that in binding assays, GBL showed highbinding affinity for GABA_A_ receptors. Additionally, after treatment with GBL, the fraction of the total time in REM and NREM significantly increased in a dose-dependent manner increased in stress-induced rats. Consistent with the behavioral results, the GBL-treated group showed a significant dose-dependent increase in the expression of GABA_A_ receptor immunopositive cells in the VLPO. Additionally, the GBL-treated group showed a significant decrease in orexin levels in the LH. Overall, GBL has shown efficacy and the potential to be used as an anti-stress agent to treat sleep disorders through the stimulation of GABA_A_ receptor and the subsequent inhibition of orexin activity. 

## 6. Limitations

In light of these limitations, our findings provide preliminary evidence for the safety of GBL and its potential to improve sleep quality in animal models. We are planning to analyze stress sleep-related neurotransmitter markers such as serotonin synthesis and release in the future study.

## Data Availability

The data used to support the findings of this study are available from the corresponding author upon request.
